# Rapid appraisal of liver diseases using transient elastography, abdominal ultrasound, and microbiology in Côte d’Ivoire: A single-center study

**DOI:** 10.1371/journal.pntd.0012262

**Published:** 2024-06-20

**Authors:** Marie T. Leibenguth, Jean T. Coulibaly, Kigbafori D. Silué, Yves K. N’Gbesso, Ahmed Abd El Wahed, Jürg Utzinger, Sören L. Becker, Sophie Schneitler

**Affiliations:** 1 Institute of Medical Microbiology and Hygiene, Saarland University, Homburg/Saar, Germany; 2 Swiss Tropical and Public Health Institute, Allschwil, Switzerland; 3 University of Basel, Basel, Switzerland; 4 Unité de Formation et de Recherche Biosciences, Université Félix Houphouët-Boigny, Abidjan, Côte d’Ivoire; 5 Centre Suisse de Recherches Scientifiques en Côte d’Ivoire, Abidjan, Côte d’Ivoire; 6 Ministère de la Santé et de l’Hygiène Publique, Centre de Santé Urbain d’Azaguié, Azaguié, Côte d’Ivoire; 7 Institute of Animal Hygiene and Veterinary Public Health, University of Leipzig, Leipzig, Germany; 8 Institute of Pneumology at the University of Cologne, Bethanien Hospital, Clinic for Pneumology and Allergology, Centre of Sleep Medicine and Respiratory Care, Solingen, Germany; University of Passo Fundo: Universidade de Passo Fundo, BRAZIL

## Abstract

**Background:**

Liver diseases of infectious and non-infectious etiology cause considerable morbidity and mortality, particularly in low- and middle-income countries (LMICs). However, data on the prevalence of liver diseases and underlying risk factors in LMICs are scarce. The objective of this study was to elucidate the occurrence of infectious diseases among individuals with chronic liver damage in a rural setting of Côte d’Ivoire.

**Methodology:**

In 2021, we screened 696 individuals from four villages in the southern part of Côte d’Ivoire for hepatic fibrosis and steatosis, employing transient elastography (TE) and controlled attenuation parameter (CAP). We classified CAP ≥248 dB/m as steatosis, TE ≥7.2 kPa as fibrosis, and did subgroup analysis for participants with TE ranging from 7.2 kPa to 9.1 kPa. Clinical and microbiologic characteristics were compared to an age- and sex-matched control group (TE <6.0 kPa; n = 109). Stool samples were subjected to duplicate Kato-Katz thick smears for diagnosis of *Schistosoma mansoni*. Venous blood samples were examined for hepatitis B and hepatitis C virus. Additionally, an abdominal ultrasound examination was performed.

**Principal findings:**

Among 684 individuals with valid TE measurements, TE screening identified hepatic pathologies in 149 participants (17% with fibrosis and 6% with steatosis). 419 participants were included for further analyses, of which 261 had complete microbiologic analyses available. The prevalence of *S*. *mansoni*, hepatitis B, and hepatitis C were 30%, 14%, and 7%, respectively. Logistic regression analysis revealed higher odds for having TE results between 7.2 kPa and 9.1 kPa in individuals with *S*. *mansoni* infection (odds ratio [OR] = 3.02, 95% confidence interval [CI] = 1.58–5.76, *P* = 0.001), while HCV infection (OR = 5.02, 95% CI = 1.72–14.69, *P* = 0.003) and steatosis (OR = 4.62, 95% CI = 1.60–13.35, *P* = 0.005) were found to be risk factors for TE ≥9.2 kPa.

**Conclusions/significance:**

Besides viral hepatitis, *S*. *mansoni* also warrants consideration as a pathogen causing liver fibrosis in Côte d’Ivoire. In-depth diagnostic work-up among individuals with abnormal TE findings might be a cost-effective public health strategy.

## Author summary

At a global scale, chronic liver diseases such as liver fibrosis or cirrhosis are increasing. Causes and risk factors vary from one setting to another. In Europe, alcohol abuse, viral hepatitis, and fatty liver disease are the leading causes. Neglected tropical diseases, especially infections with parasites such as *Schistosoma mansoni*, are known to cause liver damage in low- and middle-income countries. Precise data on the relevance of infections with *S*. *mansoni* compared to other causes of liver fibrosis are scarce. In this study, we aimed to elucidate the infectious diseases etiology of chronic liver diseases in a schistosomiasis-endemic setting of Côte d’Ivoire. We screened residents from four villages in the southern part of Côte d’Ivoire with transient elastography, a non-invasive method that detects liver fibrosis or -cirrhosis by liver stiffness. Participants were tested for schistosomiasis and viral hepatitis, and a questionnaire was used to collect sociodemographic data. We found that liver fibrosis was common (17%), with *S*. *mansoni* being the most detected pathogen (30%). Liver diseases have a high morbidity and mortality, resulting in liver cancer or gastrointestinal haemorrhage. Schistosomiasis and hepatitis C, in particular, can be treated effectively today, so a screening program with transient elastography and subsequent treatment could prevent complications of liver disease.

## Introduction

Liver diseases are a leading cause of morbidity and mortality worldwide. Indeed, more than two million deaths are registered annually and over 200 million people suffer from liver cirrhosis, the final stage of liver fibrosis (hereinafter collectively referred to as fibrosis) [1, 2]. The epidemiology and etiology of liver diseases in high-income countries (HICs) are well understood, showing a shift from alcohol-associated to non-alcoholic fatty liver disease (NAFLD) due to rising obesity and diabetes mellitus [3]. In contrast, data on the etiopathogenesis and incidence of liver diseases in low- and middle-income countries (LMICs), especially in sub-Saharan Africa, are scarce [4, 5]. Nevertheless, it is estimated that approximately 2% of deaths in sub-Saharan Africa are attributable to liver cirrhosis [2].

The diagnosis of fibrosis is complex, expensive, and, with liver puncture as the current ‘gold’ standard, invasive. Data from sub-Saharan Africa mostly referred to patients attending centers specialized in liver diseases, and hence, do not represent the general population. Research on the prevalence and risk factors of liver diseases is essential to guide preventive measures and reduce morbidity and premature death. This is particularly salient as lifestyle-related risk factors gain traction in tropical and subtropical regions, which govern non-communicable diseases (NCDs) [6]. In view of the rising incidence of NCDs, such as obesity and diabetes, the role of infectious pathogens as an additive damaging agent must be better understood, so that multifaceted prevention strategies can be implemented [6]. In sub-Saharan Africa, infections with hepatitis B virus (HBV), followed by hepatitis C virus (HCV), are considered the major cause of liver fibrosis due to their high prevalence [5]. The etiopathogenetic role of viral hepatitis in liver diseases is well known, but less attention is being paid to tropical liver-damaging pathogens, such as trematodes of the genus *Schistosoma*. Worldwide, an estimated 250 million people are infected with *Schistosoma* spp., with most cases concentrated in sub-Saharan Africa [7]. S. *mansoni* must be considered as a cause of liver damage in the context of hepatosplenic schistosomiasis. Several years after infection, if left untreated, the local hepatic inflammatory reaction caused by migrated parasite eggs can lead to periportal fibrosis and complications (e.g., variceal bleeding).

The diagnosis, treatment, and prevention of liver fibrosis are based on its etiopathogenesis. This includes providing information, education, and communication on alcohol consumption, access to antiviral therapy, and deworming campaigns for the control of schistosomiasis and other helminth infections. The variety of possible causes of liver fibrosis renders blood and stool screening in the general population unfeasible and costly. Transient elastography (TE) is a technique that allows the rapid and non-invasive detection of liver fibrosis using shear wave diagnostics [8]. New devices allow the simultaneous measurement of controlled attenuation parameter (CAP) to detect steatosis. TE and CAP screening might provide an alternative approach for identifying individuals at risk, particularly in at-risk populations or regions with high prevalence rates of liver diseases [8].

The study reported here was conducted in a *Schistosoma*-endemic area in the southern part of Côte d’Ivoire. In 2010, the prevalence of *S*. *mansoni* infections in children in the area was found to be higher than 90% [9,10]. Data on the epidemiology of liver cirrhosis or viral hepatitis in the district were not available prior to our study. The overall HBV prevalence in the general population of Côte d’Ivoire is estimated to be approximately 8% and the HCV prevalence is estimated to be around 1% [11]. In this study, we screened the population for liver fibrosis and estimated the prevalence of liver diseases [9,10]. Participants identified with liver disease and an age- and sex-matched control group were further investigated to determine risk factors for liver diseases based on an ensemble of medical history, ultrasound, and microbiologic examinations.

## Methods

### Ethics statement

Ethical approval for this study was obtained from the ‘Comité National d’Ethique des Sciences de la Vie et de la Santé’ (CNESVS; Abidjan, Côte d’Ivoire; reference no. 039–21) and from the ethics committee at the ‘Ärztekammer des Saarlandes’ (Saarbrücken, Germany; reference no. 246/20). The individual examination results were communicated confidentially to each participant. All participants who were found to have an underlying disease (e.g., infection, ascites) were actively referred to the Azaguié Municipal Health Center for further diagnosis and treatment. Patients infected with *S*. *mansoni* received praziquantel.

### Study area

The study was carried out in Azaguié district in the southern part of Côte d’Ivoire. The area is known to be endemic for schistosomiasis [9,10]. Participants were recruited from four villages (i.e., Ancien Carrefour, M’Bromé, Odoguié, and Elevi).

### Design and recruitment

The study was designed as a cross-sectional study. In February 2021, a door-to-door census was carried out and a list was generated with all residents of the four villages aged 18 years and above. Throughout the census process, participants received comprehensive briefings regarding the study’s objectives, methodologies, and potential risk and benefits. A community-wide informational event was convened, and all residents were invited to voluntarily engage in the study. Additionally, healthcare professionals diligently interacted with residents, imparting detailed information concerning the study and its implications.

The study was conducted either in rooms provided by the village community or in rooms in the health centers. However, participants were not recruited at the health center; instead, residents who wished to participate in the study were asked to report to the site on one of the specific two to three days (depending on the village) during which the study team was present. All participants were informed about the purpose and procedures of the study, including potential risks and benefits. Written informed consent was provided and participants could withdraw at any time without further obligations. Pregnant women and residents who had taken deworming medication up to 4 weeks previously were excluded.

### Procedures

Anthropometric data were collected, including blood pressure, height, weight, heart rate, body temperature (Braun NTF 3000; Lausanne, Switzerland), and abdominal circumference. These data were measured only once at the time of enrolment in the study. Participants underwent TE and CAP screening for liver fibrosis and steatosis using FibroScan Mini+ (Echosens; Paris, France). To measure stiffness in the right liver lobe, the probe was placed intercostally at the level of the xiphoid process in the midclavicular line. According to general recommendations, a TE result was only accepted if 10 valid measurements were taken. In case of a result ≥7.2 kPa, which indicates liver disease, the interquartile range (IQR) had to be ≤30%. Otherwise, the measurement was repeated.

Participants with a TE result of ≥6.0 kPa were included for further investigation (ultrasound, blood and stool examinations). Additionally, we included an age- and sex-matched control group (<6.0 kPa). For this purpose, after the inclusion of a participant, the next screened participant with the same age and sex, but with a TE result of <6.0 kPa, was included in the control group. A TE result of 6.0–7.1 kPa was considered as ‘possible liver fibrosis’, while a TE result of ≥7.2 kPa was deemed as ‘liver fibrosis’. This dual cut-off strategy allowed the exclusion of fibrosis with a high negative predictive value using the first cut-off, while the second cut-off corresponds to the optimal value for fibrosis detection [12, 13]. The liver fibrosis group was analyzed in subgroups with a “low” (7.2–9.1 kPa) and “high” cut-off (≥9.2 kPa) group to account for different cut-offs depending on the underlying etiology. CAP values ≥248 dB/m were classified as steatosis [14]. All screened participants underwent TE measurement, CAP measurement, and anthropometric assessment. Only those with TE >6.0 were included in subsequent examinations (e.g., ultrasound and laboratory investigations, as described below) along with the control group.

The TE and CAP examination was followed by a detailed ultrasound examination of the abdomen, whereby both the examiner and the participant were blinded to the TE results. Data were collected using a standardized ultrasound protocol, with an emphasis on fibrosis signs according to the Niamey protocol for sonographic evaluation of chronic schistosomiasis [15]. Participants were interviewed by trained health workers using a questionnaire regarding the presence of symptoms and risk factors of liver disease.

### Laboratory investigations

A venous blood sample was taken from the cubital vein and a blood count was done, including alanine aminotransferase (ALT), and bilirubin measurements. Serology was performed to detect hepatitis B surface antigen (HBsAg) (Liaison HBsAg, Diasorin; Saluggia, Italy) and hepatitis C antibodies (anti-HCV) (Murex anti-HCV, Diasorin; Saluggia, Italy). Participants with sufficiently large blood samples were also tested for antibodies against hepatitis A (Liaison anti-HAV, Diasorin; Saluggia, Italy) and hepatitis E (Anti Hepatitis E Virus (HEV) ELISA IgG, Euroimmun; Lübeck, Germany).

Participants were invited to provide two consecutive stool samples on the days following their examination. Stool samples were analyzed for the presence of parasite eggs using the Kato-Katz technique. Duplicate Kato-Katz thick smears, using standard 41.7 mg templates, were examined under a microscope by experienced laboratory technicians [16]. The number of eggs counted per slide were multiplied by a factor of 24 to obtain a measure of infection intensity, expressed by eggs per gram of stool (EPG). Intensity of *S*. *mansoni* infection was classified as mild (1–99 EPG), moderate (100–399 EPG), and heavy (≥400 EPG), according to guidelines put forth by the World Health Organization (WHO) [17].

### Statistical analysis

Data were double-entered and cross-checked in Excel (version 2201) and analyzed with IBM SPSS statistics (version 27). Group differences were assessed for qualitative variables by Pearson’s χ^2^ or Fisher’s exact test, as appropriate. Quantitative variables were analyzed using a Kruskal-Wallis test. Risk factors for liver fibrosis were determined with logistic regression models. For all plausible risk factors for fibrosis, univariable logistic regression was used to determine the crude odds of having a TE result ranging from 7.2–9.1 kPa and >9.1 kPa. Variables that were not considered risk factors for fibrosis but might be associated with an elevated risk for (previous) infections with viral hepatitis or *S*. *mansoni* were also included. All risk factors associated with liver fibrosis with a significance <0.1 in the univariable analysis were included in the multivariable analysis, stratified by sex, age, and residency.

## Results

### Census and TE screening results

**Fig 1:**
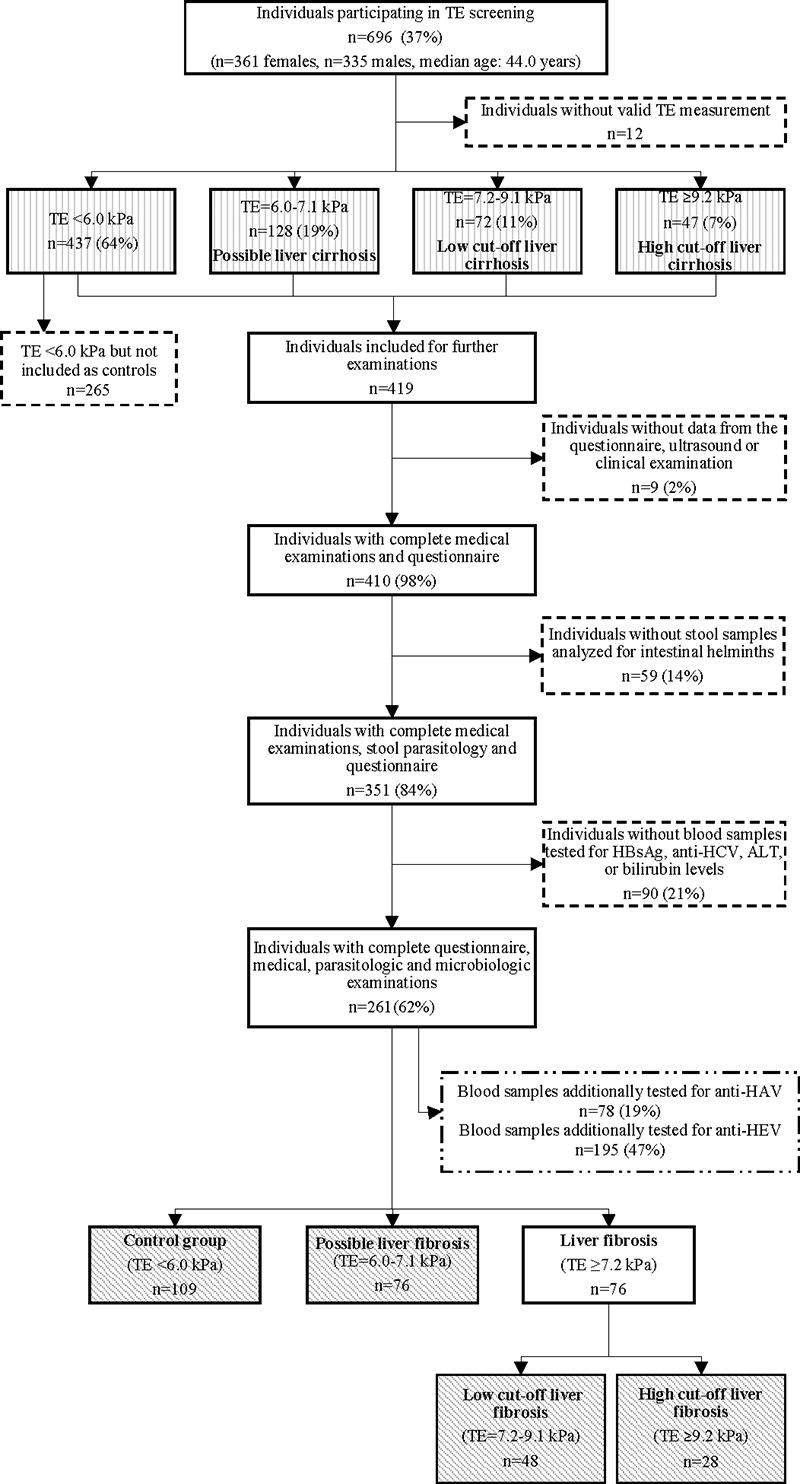
Flowchart for a study investigating transient elastography (TE) as a screening tool for liver disease in the southern part of Côte d’Ivoire, March 2021. The boxes with vertical lines indicate the participants who took part in the screening and were included in Table 1. Based on TE measurements, participants were stratified as “liver fibrosis” (high- or low cut-off), “possible liver fibrosis”, or “control group” (without liver fibrosis) and were subjected to a host of additional diagnostics for infectious diseases. The groups included in Tables 2 and 3 are shown in the boxes with diagonal shading.

A total of 1,880 residents aged ≥18 years were registered (median age 40 years, 49% female). Between 15 and 26 March 2021, 696 residents (37%), aged 18–95 years (median age 44 years, 52% female), were screened for liver diseases using TE. Twelve participants were excluded because of invalid TE measurements (Fig 1). On average, an individual examination took 2.2 min.

Two-third of the participants (n = 437, 64%) had a TE result <6.0 kPa (no fibrosis). There were 128 participants (19%) with a TE result between 6.0 kPA and 7.1 kPa, and 119 participants (17%) had TE results ≥7.2 kPa (fibrosis). Males were significantly more likely to have fibrosis than females (21% [70/330] vs. 14% [49/354], *P* = 0.002). Females were twice as likely to have steatosis than males (8% [29/354] vs. 4% [13/330], *P* = 0.020). Body mass index (BMI) was significantly higher in females compared to males (Table 1). Both fibrosis (*P* <0.001) and steatosis (*P* = 0.002), were associated with higher systolic blood pressure.

**Table 1:** Epidemiologic baseline data and characteristics of individuals participating in the TE screening in Azaguié, in the southern part of Côte d’Ivoire, March 2021.

Variable	Sex		Age group (years)				All
	Female (n = 354)	Male (n = 330)	18–29 (n = 120)	30–44 (n = 235)	45–59 (n = 205)	≥60 (n = 124)	(n = 684)
**TE mean (kPa)**	5.8 (4.6)	6.3 (2.9)	5.8 (2.6)	6.4 (5.3)	6.1 (3.4)	5.7 (1.8)	6.1 (3.8)
<6.0	248 (70)	189 (57)	84 (70)	139 (59)	133 (65)	81 (65)	437 (64)
6.0–7.1	57 (16)	71 (22)	18 (15)	47 (20)	42 (20)	21 (17)	128 (19)
7.2–9.1	32 (9)	40 (12)	13 (11)	30 (13)	14 (7)	15 (12)	72 (11)
≥9.2	17 (5)	30 (9)	5 (4)	19 (8)	16 (8)	7 (6)	47 (7)
**CAP mean (dB/m)**	197.7 (43.7)	191.1 (32.4)	178.9 (32.6)	193.6 (37.9)	204.9 (38.6)	194.3 (41.2)	194.5 (38.8)
Steatosis (CAP ≥ 248)	29 (8)	13 (4)	0 (0)	15 (6)	19 (9)	8 (6)	42 (6)
BMI (kg/m^2^)	22.7 (4.3)	21.5 (3.0)	21.3 (2.8)	22.3 (3.7)	22.9 (4.4)	21.4 (3.4)	22.1 (3.8)
Overweight (BMI ≥ 25)	92 (26)	30 (9)	12 (10)	42 (18)	46 (22)	22 (18)	122 (18)
Underweight (BMI < 18.5)	54 (15)	33 (10)	22 (18)	23 (10)	19 (9)	23 (19)	87 (13)
Abdominal circumference (cm)	80.2 (10.9)	77.7 (7.8)	73.7 (7.4)	79.0 (9.0)	81.5 (9.1)	80.0 (10.5)	79.0 (9.6)
**Systolic blood pressure (mmHg)**	132.3 (27.3)	136.8 (23.3)	120.7 (17.9)	126.8 (18.7)	140.1 (26.0)	153.1 (28.7)	134.5 (25.5)
Hypertension (sys. > 140)	112 (32)	124 (38)	13 (11)	54 (23)	93 (45)	76 (62)	236 (35)

Data are mean (standard deviation) or n (%), unless otherwise specified; percentage of TE classification refers to sex and age group; (TE = transient elastography, CAP = controlled attenuation parameter; BMI = body mass index; sys. = systolic blood pressure)

### Comparative characteristics of participants with or without liver fibrosis

The full results of all further investigations were available for 261 of 419 (62%) included participants (152 with TE ≥6.0 kPa and 109 with TE <6.0 kPa). There were no significant associations regarding social circumstances, e.g., income, educational attainment, and the occurrence of liver fibrosis (Table 2). In the high cut-off fibrosis group, 21% [6/28] had steatosis (vs. 3% [3/109] of the control group; *P* = 0.011). Compared to the control group, those with liver fibrosis were about twice as likely to be over- or underweight.

**Table 2:** Demographic and clinical characteristics of participants in a study on transient elastography (TE)-based screening for detection of hepatic diseases in Azaguié, in the southern part of Côte d’Ivoire, March 2021. Data are stratified by TE measurements as “controls”, “possible liver fibrosis”, and “liver fibrosis” (subgroup analysis for low [7.2 kPa] and high [9.2 kPa] cut-off) (n (%)).

Variable (n = 261)	Controls (<6.0 kPa) (n = 109)	Possible liver fibrosis (6.0–7.1 kPa) (n = 76)	Liver fibrosis (7.2–9.1 kPa) (n = 48)	Liver fibrosis (≥9.2 kPa) (n = 28)	Entire study population (n = 261)	*P*
**Age (years)**	46.7 (12.3)	46.8 (13.9)	43.6 (16.9)	46.4 (12.3)	46.1 (13.7)	0.270
**Sex**						
Female	49 (45)	39 (51)	23 (48)	13 (46)	124 (48)	0.863
Male	60 (55)	37 (49)	25 (52)	15 (54)	137 (52)	
**Educational attainment**						0.293*
No formal school education	36 (33)	30 (39)	19 (40)	8 (29)	93 (36)	
Primary school	39 (36)	34 (45)	15 (31)	12 (43)	100 (38)	
Secondary school	23 (21)	11 (14)	9 (19)	6 (21)	49 (19)	
Higher education	11 (10)	1 (1)	5 (10)	2 (7)	19 (7)	
**Household income (n = 242)**						0.967*
<45 US$/month	41 (40)	30 (44)	22 (48)	11 (42)	104 (43)	
45–145 US$/month	49 (48)	28 (41)	19 (41)	12 (46)	108 (45)	
>145 US$/month	12 (12)	10 (15)	5 (11)	3 (12)	30 (12)	
**Occupation**						0.433*
Trader	14 (13)	13 (17)	5 (10)	2 (7)	34 (13)	
Farmer	69 (63)	43 (57)	28 (58)	18 (64)	158 (61)	
Other	14 (13)	10 (13)	13 (27)	5 (18)	42 (16)	
Unemployed	12 (11)	10 (13)	2 (4)	3 (11)	27 (10)	
**Marital status (n = 256)**						0.229*
Single	16 (15)	19 (26)	14 (29)	3 (11)	52 (20)	
Married	80 (74)	44 (60)	29 (60)	21 (78)	174 (68)	
Widowed	12 (11)	10 (14)	5 (10)	3 (11)	30 (12)	
**Health status**						
Underweight (BMI < 18.5 kg/m^2^)	12 (11)	10 (13)	11 (23)	4 (14)	37 (14)	0.276*
Overweight (BMI ≥ 25 kg/m^2^)	11 (10)	17 (22)	10 (21)	5 (18)	43 (16)	0.097*
Steatosis (CAP ≥ 248 dB/m)	3 (3)	6 (8)	4 (8)	6 (21)	19 (7)	0.011*
Tachycardia (HR > 100 bpm)	1 (1)	7 (9)	6 (13)	4 (14)	18 (7)	0.002*
Hypertension (sys. >14.0 mmHg)	41 (38)	29 (38)	15 (31)	15 (54)	100 (38)	0.285
**Laboratory results**						
Elevated conjugated bilirubin (>5 mg/l)	3 (3)	1 (1)	6 (13)	3 (11)	13 (5)	0.009*
Elevated ALT (f: > 35 U/l; m: > 50 U/l)	2 (2)	2 (3)	3 (6)	5 (18)	12 (5)	0.006*
Platelet count (<150 10^3^/mm^3^)	1 (1)	0 (0)	1 (2)	3 (11)	5 (2)	**0.010**
Anemia (f: Hb < 12 g/dl; m: Hb < 13 g/dl)	32 (30)	19 (29)	15 (33)	12 (44)	78 (32)	0.488
**Medical history**						
Diarrhea (last 6 months)	25 (23)	22 (29)	11 (23)	7 (25)	65 (25)	0.803
Abdominal pain (last 6 months)	33 (30)	29 (38)	18 (38)	11 (39)	91 (35)	0.623
Hospitalization (last 6 months)	2 (2)	1 (1)	2 (4)	2 (7)	7 (3)	0.421*
Blood transfusion (ever received)	6 (6)	1 (1)	4 (8)	3 (11)	14 (5)	0.120*
Piercing (ever had)	12 (11)	6 (8)	10 (21)	3 (11)	31 (12)	0.217*
Regular alcohol consumption (at least weekly)	23 (21)	18 (24)	10 (21)	7 (25)	58 (22)	0.982*

Data are mean (standard deviation) or n (%), unless otherwise specified

*Fisher’s exact test; (CAP = controlled attenuation parameter; BMI = body mass index; HR = heart rate; sys. = systolic blood pressure; ALT = alanine aminotransferase; Hb = hemoglobin; f = female; m = male)

Only two of the participants with liver fibrosis reported having a liver disease in the questionnaire survey. No participant reported ever having received any treatment for viral hepatitis. Eleven participants stated that they had ever had a *Schistosoma* infection.

### Prevalence of different pathogens giving rise to hepatic diseases in participants with or without liver fibrosis

The prevalence of *S*. *mansoni* infection, HBV, and HCV were 30%, 14%, and 7%, respectively (Table 3). There were no significant sex differences regarding the prevalence of any of these infections.

**Table 3:** Prevalence of different pathogens giving rise to hepatic diseases in participants without liver fibrosis as compared to individuals with “possible fibrosis” and “fibrosis”, as derived from transient elastography in Azaguié, in the southern part of Côte d’Ivoire, March 2021.

Variable	Controls (<6.0 kPa) (n = 109)	Possible fibrosis (6.0–7.1 kPa) (n = 76)	Liver fibrosis (7.2–9.1 kPa) (n = 48)	Liver fibrosis (≥9.2 kPa) (n = 28)	Entire study population (n = 261)^a^^,^^b^	*P*
***Schistosoma mansoni***	24 (22)	20 (26)	24 (50)	9 (32)	77 (30)	**0.004**
**HAV** ^**a**^	27 (90)	25 (93)	12 (100)	9 (100)	73 (94)	0.914*
**HBV**	13 (12)	10 (13)	9 (19)	5 (18)	37 (14)	0.602*
**HCV**	7 (6)	2 (3)	3 (6)	6 (21)	18 (7)	**0.020** *
**HEV** ^**b**^	19 (23)	16 (27)	8 (22)	6 (38)	49 (25)	0.606*
**Infection with at least one hepatic pathogen (*S*. *mansoni*, HBV, HCV)** **Coinfections:**	40 (37)	29 (38)	30 (63)	18 (64)	117 (45)	**0.002**
**- *S*. *mansoni*/HBV coinfection**	4 (4)	3 (4)	4 (8)	1 (4)	12 (5)	0.582*
**- *S*. *mansoni*/HCV coinfection**	0 (0)	0 (0)	2 (4)	1 (4)	3 (1)	**0.035** *
**- HBV/HCV coinfection**	0 (0)	0 (0)	1 (2)	0 (0)	1 (0)	0.293*
**- *S*. *mansoni*/HBV/HCV triple infection**	0 (0)	0 (0)	1 (2)	0 (0)	1 (0)	0.293*

n (%)

*Fisher’s exact test

^a^ Test results for hepatitis A only available for 78 participants

^b^ Test results for hepatitis E only available for 195 participants

Abbreviations: HAV: hepatitis A virus (anti-HAV-antibodies positive); HBV: hepatitis B Virus (HbsAg positive); HCV: hepatitis C virus (anti-HCV-antibodies positive); HEV: hepatitis E virus (anti-HEV antibodies positive)

Half of the low cut-off fibrosis group was infected with *S*. *mansoni* [24/48], compared to 32% of the high cut-off group [9/28] and 22% of the controls [22/109] (*P* = 0.004). Regarding the infection intensity, there were no significant differences between the four groups. Only 4 of 77 infections (5%) were of heavy intensity; none in the control group [0/22]. Individuals with *S*. *mansoni* infection were significantly younger than those without (43.6 vs. 47.2 years, *P* = 0.018). In comparison to other participants with fibrosis, individuals infected with *S*. *mansoni* had lower mean TE (8.9 kPa vs. 10.2 kPa, *P* = 0.017) and CAP (184.8 dB/m vs. 212.4 dB/m, *P* = 0.007).

Regarding viral hepatitis, anti-HCV was detected in 21% of the high cut-off group, compared to 6% in both the low cut-off and control group. In contrast to *S*. *mansoni*, the HCV-positive participants were older than the non-infected counterparts (52.3 vs. 45.7 years, *P* = 0.048). Only 3 of 261 participants (1%) were coinfected with HCV and *S*. *mansoni*; all having liver fibrosis. The HBV prevalence was similar in the high and low cut-off groups and slightly higher in those with liver fibrosis than in the control group (18% vs. 12%, *P* = 0.602). Hepatitis A and E antibodies were detected in 94% [73/78] and 25% [49/195] of participants with available test results, respectively, as evidence of previous exposure to the pathogen. There were no significant associations with TE and CAP results.

In the univariable logistic regression analysis, the presence of *S*. *mansoni* infection was found to be a risk factor for having TE results between 7.2 kPa and 9.1 kPa, while HCV infection and having steatosis were found to be risk factors for TE results ≥9.2 kPa (Table 4). For female participants, multivariable regression analysis revealed low household income (<45 US$/month) as an additional risk factor for fibrosis (adjusted odds ratio [OR] = 2.80, 95% confidence interval [CI] 1.09–7.20, *P* = 0.032).

**Table 4:** Univariable and multivariable logistic regression analysis on the association of different epidemiologic, clinical, and infectious disease characteristics in a study on liver diseases in Azaguié, in the southern part of Côte d’Ivoire, March 2021.

Variable		Univariable regression 7.2–9.1 kPa		≥9.2 kPa		Multivariable regression ≥7.2 kPa	
Risk factor	n	OR (95% CI)	*P*	OR (95% CI)	*P*	OR (95% CI)	*P*
***S*. *mansoni***	77	3.02 (1.58–5.76)	**0.001**	1.15 (0.50–2.67)	0.746	2.93 (1.58–5.43)	**<0.001**
**HBs-Ag**	37	0.66 (0.29–1.50)	0.317	0.73 (0.26–2.06)	0.556		
**Anti-HCV**	18	0.88 (0.24–3.17)	0.845	5.02 (1.72–14.69)	**0.003**	3.92 (1.39–11.11)	**0.010**
**Blood transfusion**	14	1.85 (0.55–6.15)	0.319	2.42 (0.63–9.27)	0.196		
**Regular alcohol consumption (at least weekly)**	58	0.52 (0.15–1.81)	0.306	1.51 (0.48–4.75)	0.477		
**Known liver disease in family**	5	1.11 (0.12–10.17)	0.925	2.12 (0.23–19.67)	0.508		
**Overweight (BMI ≥25 kg/m** ^**2**^ **)**	43	1.44 (0.65–3.16)	0.369	1.12 (0.40–3.12)	0.835		
**Steatosis (CAP ≥248 dB/m)**	19	1.2 (0.38–3.79)	0.756	4.62 (1.60–13.35)	**0.005**	3.49 (1.27–9.56)	**0.015**
**Smoking**	36	1.32 (0.56–3.11)	0.524	1.05 (0.34–3.22)	0.936		
**Dilated hepatic veins**	40	1.13 (0.48–2.64)	0.775	1.6 (0.60–4.22)	0.346		
**Household size: more than 6 people**	115	0.8 (0.42–1.51)	0.490	0.68 (0.30–1.53)	0.349		
**Household income: <45 US$/month**	104	1.35 (0.72–2.54)	0.349	0.97 (0.44–2.17)	0.949		
**No toilet at home**	94	0.87 (0.45–1.68)	0.668	1.38 (0.62–3.06)	0.426		
**Fresh water contact during: bathing**	66	0.98 (0.48–2.02)	0.960	0.46 (0.15–1.38)	0.165		
**Water contact: never/rarely**	46	0.92 (0.40–2.13)	0.847	1.32 (0.50–3.45)	0.577		
**No school education**	93	1.23 (0.65–2.34)	0.527	1.44 (0.61–3.40)	0.411		
**Profession: farmer**	158	0.89 (0.47–1.69)	0.730	1.2 (0.53–2.70)	0.668		

Shown are the results from univariate and multivariate logistic regression analysis, controlling for age, sex, and residency (CI: confidence interval; OR: odds ratio)

In a univariable subgroup analysis of the *S*. *mansoni*-positive participants, the infection intensity of *S*. *mansoni* and low household income were significantly associated with liver damage in those participants, with only the low household income as an independent risk factor in the multivariable regression model (adjusted OR = 5.40, 95% CI 1.60–18.25, *P* = 0.007).

On abdominal ultrasound, one in five participants had sonographic signs of periportal fibrosis (S1 File). In 41% of these participants, infection with *S*. *mansoni* could be proven in the stool examination. The median age of participants with periportal fibrosis was 45 years and the mean age 48 years (standard deviation: 15 years). In contrast, participants who were infected with *S*. *mansoni* but did not have periportal fibrosis on ultrasound had a median age of 40 years (mean age: 44 years, standard deviation: 16 years). 43% of those who had liver fibrosis in TE (high cut-off) showed signs of periportal fibrosis on ultrasound. 46% of those who were HCV negative and had high cut-off liver fibrosis in TE showed signs of periportal fibrosis on ultrasound.

## Discussion

The screening revealed a high prevalence of liver diseases in four villages in the district of Azaguié in the southern part of Côte d’Ivoire. Risk factors for liver damage in the studied population were, besides male sex, infections with *S*. *mansoni*, HCV, and steatosis. Low household income was identified as a risk factor for females or in case of *S*. *mansoni* infection. We found that TE is a suitable tool for liver screening in the general population in areas with limited healthcare infrastructure.

The most prevalent risk factor for liver diseases was infection with *S*. *mansoni*, which was particularly evident in the younger population of our study (aged 18–29 years). However, since more than one fifth of the control group was also infected with *S*. *mansoni*, it remains unclear to what extent *S*. *mansoni* is driving liver damage, including underlying risk factors [18]. In our study, a lower household income was the only associated risk factor. It is worth noting that the participants with pipe stem fibrosis were, on average, older than those with *S*. *mansoni* infection. As the infection precedes the fibrosis, it is possible that younger participants may be infected but have not yet developed fibrosis. The lower median age of participants infected with *S*. *mansoni* compared to those with pipe stem fibrosis provides additional evidence of the causal relationship between *S*. *mansoni* and liver fibrosis in this population.

*S*. *mansoni*-related liver fibrosis is a long-term consequence and can be prevented by early treatment with praziquantel. Furthermore, praziquantel can reduce already existing liver fibrosis [19]. In many schistosomiasis-endemic settings, effective mass deworming programs focus primarily on school-age children. However, although community-based programs exist, the treatment of adults has been less comprehensive, which must be improved and sustained to prevent long-term complications [20]. As a neglected tropical disease, there is too little focus on schistosomiasis as a cause of liver damage and portal hypertension. Screening programs for liver fibrosis must identify those at highest risk to prevent life-threatening complications, particularly variceal bleeding. On the other hand, the treatment of periportal fibrosis as a specific condition needs to be further investigated and standardized. To date, most therapies for periportal fibrosis are adapted to therapies of other liver diseases, and hence, lack evidence regarding their effectiveness for hepatosplenic schistosomiasis [21].

Viral hepatitis is generally regarded as the leading risk factor for liver fibrosis in Africa, with HBV being more common than HCV [5]. Our study provides new evidence on viral hepatitis in the general population in a rural setting of in the southern part of Côte d’Ivoire. There is a paucity of data, as many studies only focused on high-risk groups (e.g., HIV patients) and did not screen at the community level. In our study, as expected, HBV was more prevalent than HCV. HBV caused liver damage in fewer infected individuals than HCV and *S*. *mansoni*. Future studies should also verify the determined HCV and HBV prevalences with confirmation by polymerase chain reaction (PCR) to rule out whether previous infections have been cured and determine the viral load and predominant genotype, which influences the severity of liver damage and the proportion of asymptomatic HBsAg carriers [22,23].

Regarding viral hepatitis, it is vital to make appropriate diagnostics accessible. None of the infected study participants knew about their infection, which entails a risk of infection for others. For hepatitis C, the treatment with direct antiviral agents has been a turning point in therapy, with a >95% success rate of cure [24]. Given the high costs of therapy, it might be possible to use TE to select those patients who are eligible for treatment.

In addition to infectious agents, NAFLD is a risk factor due to the increasing incidence of obesity and diabetes in African countries [25]. To date, there is limited reliable data on NAFLD in sub-Saharan Africa, as it can also be assessed either by invasive biopsy, or by ultrasound, which provides poor comparability of results. In this study, we screened the population using CAP, which provides a comparable and objective assessment of steatosis. Females were particularly affected, with approximately 1 in 12 women having steatosis. Steatosis of both alcoholic and non-alcoholic origin can develop into steatohepatitis and cirrhosis. The presence of other risk factors such as viral hepatitis may also lead to an aggravation of liver damage. *S*. *mansoni*-infected participants in our study had lower mean CAP results, but the consequences of hepatosplenic schistosomiasis in the presence of NAFLD have yet to be fully investigated.

It has been proven for the etiopathogenesis of NAFLD that even though lifestyle-dependent factors have a considerable influence, the occurrence of the disease also depends decisively on genetic disposition. Some studies showed that African Americans, even with higher risk profiles for NAFLD, had lower steatosis values compared to Caucasians [26,27]. The extent to which genetic and ethnic differences play a role in liver disease in the African region needs to be investigated, as well as the potential impact of ethnic differences on CAP and TE measurements and associated thresholds.

Our study has several limitations. First, the study population was relatively small, and some participants had an insufficient volume of the blood sample taken to perform all tests. Although we were able to screen a large proportion of the population of the four villages (37%), the average age of the screened participants and the proportion of females were slightly higher than of the overall population, which may have introduced some bias. Larger multicenter studies are warranted that should take into account seasonal and local variations in infection. Second, TE cut-offs differ depending on the etiology of the liver damage. In our subgroup analysis, we followed the recommendation of a cut-off of 9.2 kPa for population-based screenings, but there are still no standardized cut-offs for schistosomiasis-endemic regions [8]. Recent studies suggest cut-offs between 6.1 kPa and 8.0 kPa for hepatosplenic schistosomiasis and, as in our study, it was shown that patients with hepatosplenic schistosomiasis have lower mean TE than fibrosis patients of other etiologies [28–30]. Based on our observations, slightly elevated TE results could possibly detect even minor damage due to infection than sonographic proof of periportal fibrosis. However, increased TE values indicate portal hypertension and esophageal varices. Hence, further studies are needed to establish precise cut-offs for detecting fibrosis in schistosomiasis and patients at acute risk of variceal bleeding [31]. Third, the cost-effectiveness of the TE screening in LMICs must be assessed in future studies. For European countries and Hong Kong, it could be shown that TE screening in the general population is highly cost-effective [32]. Considering the high prevalence of liver fibrosis and liver-damaging pathogens in the current study in Côte d’Ivoire, the potential benefit and need for such screening in schistosomiasis-endemic countries may be even higher, as a larger proportion of the population is potentially at risk. Blood and stool testing of the population for HBV, HCV, and schistosomiasis is expensive, but limiting in-depth diagnostic work-up to individuals with abnormal TE findings might be a cost-effective public health strategy. Fourth, it was beyond the scope of the current study to provide an in-depth investigation of the differential contribution of viral hepatitis to chronic liver damage. While some of our results were quite unexpected (e.g., lack of association between liver damage and HBsAg detection or absence of HCV infection in mild fibrosis), future studies are warranted to conduct, for example, additional PCR-based testing, genotype distribution assessments, and longitudinal monitoring to accurately describe the evolution of chronic viral hepatitis in this population, which may differ from patterns observed in HICs. It is worth mentioning that a HBV vaccination program for children was introduced in Côte d’Ivoire in 2000 as part of the Expanded Program on Immunisation (EPI) [33]. However, most of the adults included in our study did not benefit from this EPI as they were older than the vaccinated population at the time of its implementation. However, this could lead to a decline in the prevalence of HBV in the coming years and therefore a further reduction in its contribution to liver fibrosis.

In about one-third of the participants diagnosed with liver fibrosis in TE and ultrasound, neither steatosis nor chronic viral hepatitis or schistosomiasis was detected. The contribution of schistosomiasis to chronic liver damage might have been underestimated, as the Kato-Katz technique has limited sensitivity and can only detect active, but not previous infections. Future studies should employ additional diagnostic assays, such as circulating anodic antigen (CAA) for schistosomiasis. Further biochemical testing and liver function tests would have been desirable but were not available in the study setting. Confounding factors, such as congestive heart disease, or highly elevated ALT levels, can produce false positive TE results [12]. Our study did not examine the role of aflatoxins as a risk factor, which is considered to be a frequent cause of liver damage in African countries. Other causes, like alcohol consumption, herbal medicines, autoimmune reactions, or less frequent genetic diseases might also have caused liver damage in some participants [34]. Care is indicated with responses to questions pertaining to alcohol consumption, as participants might not have answered with full honesty. However, most of the causes of liver damage found are preventable.

To our knowledge, this study is among the first pertaining to fibrosis and steatosis screening using TE and CAP in the general population in sub-Saharan Africa, including underlying risk factors. The identified risk factors for liver fibrosis in our study area might guide the development of appropriate preventive measures. While challenging, determining the prevalence and causes of liver diseases in sub-Saharan Africa is warranted. Marginalized populations who cannot afford preventive measures are at an elevated risk. TE allows for rapid, non-invasive screening of liver fibrosis in LMICs. The cost-effectiveness of TE screening in areas where schistosomiasis and viral hepatitis are co-endemic needs to be investigated. As lifestyle-associated morbidities are likely to increase, steatosis will rise as another risk factor. It will intensify the existing burden of liver diseases, which calls for implementation of targeted prevention and treatment programs.

## Supporting information

S1 File Results of the abdominal ultrasound examination. (DOCX)
