# Supplementary material for: Rapid appraisal of liver diseases using transient elastography, abdominal ultrasound, and microbiology in Côte d’Ivoire: A single-center study
Source: PLoS Negl Trop Dis. 2024 Jun 20;18(6):e0012262. doi: 10.1371/journal.pntd.0012262 (PMC11218973; doi:10.1371/journal.pntd.0012262)
Supplement: S1 File — (DOCX) [file pntd.0012262.s001.docx]

**Supplementary material 1**

**Results of the abdominal ultrasound examination**

| Sonographic findings | Controls  (<6.0k Pa) | Possible fibrosis  (6.0-7.1 kPa) | Liver  fibrosis  (7.2-9.1 kPa) | Liver  fibrosis  (≥9.2 kPa) | Entire study population | *P* |
| --- | --- | --- | --- | --- | --- | --- |
| Hepatomegaly | 8 (9) | 8 (12) | 7 (19) | 4 (18) | 27 (13) | 0.278 |
| Liver lesions | 9 (8) | 5 (7) | 6 (13) | 5 (18) | 25 (10) | 0.283 |
| Heterogenic hepatic parenchyma and/or surface nodularity | 6 (6) | 22 (29) | 18 (38) | 17 (61) | 63 (24) | **<0.001** |
| Periportal fibrosis | 19 (17) | 10 (13) | 13 (27) | 12 (43) | 54 (21) | **0.005** |
| Dilated hepatic veins (>7 mm) | 14 (13) | 12 (16) | 8 (17) | 6 (21) | 40 (15) | 0.661 |
| Dilated splenic vein (>12 mm) | 1 (1) | 1 (1) | 1 (2) | 4 (14) | 7 (3) | **0.006** |
| Portal vein blood-flow velocity <15 cm/s (n=242) | 14 (14) | 12 (17) | 7 (16) | 10 (38) | 43 (18) | 0.050 |
| Portosystemic collateral circulation | 0 (0) | 1 (1) | 0 (0) | 2 (7) | 3 (1) | **0.023** |
| Dilated biliary ducts | 5 (5) | 6 (8) | 2 (4) | 3 (11) | 16 (6) | 0.487 |
| Splenomegaly | 17 (16) | 13 (17) | 12 (25) | 7 (26) | 49 (19) | 0.429 |
| Spleen lesions | 5 (5) | 2 (3) | 3 (6) | 2 (7) | 12 (5) | 0.602 |
| Ascites | 1 (1) | 1 (1) | 0 (0) | 1 (4) | 3 (1) | 0.509 |

Data are n (%)
